# Conservative Management and Safe Discontinuation of Continuous Antibiotic Prophylaxis in a Child With an Ectopic Ureterocele: An Eight-Year Follow-Up Case Report and Literature Review

**DOI:** 10.7759/cureus.95418

**Published:** 2025-10-26

**Authors:** Takanori Mochizuki, Norifumi Sawada, Anna Kobayashi, Miwa Goto, Takahiko Mitsui

**Affiliations:** 1 Department of Urology, Faculty of Medicine, Graduate Faculty of Interdisciplinary Research, University of Yamanashi, Chuo, JPN; 2 Department of Pediatrics, Faculty of Medicine, Graduate Faculty of Interdisciplinary Research, University of Yamanashi, Chuo, JPN

**Keywords:** conservative management, continuous antibiotic prophylaxis, ectopic ureterocele, hydronephrosis, long-term follow-up

## Abstract

An ectopic ureterocele is a rare congenital anomaly often managed surgically due to risks of urinary tract infections (UTIs), hydronephrosis, and renal impairment. Yet there is no consensus on selecting surgical or conservative observation therapy. A girl was diagnosed antenatally with a left ectopic ureterocele. Postnatal evaluation confirmed hydronephrosis, and at three months of age, voiding cystourethrography demonstrated the ureterocele without vesicoureteral reflux. At the routine six-month ultrasound, the ureterocele was noted to be smaller than at diagnosis. A follow-up magnetic resonance urography and mercaptoacetyltriglycine scintigraphy at eight months revealed further reduction in ureterocele size, improvement in hydronephrosis, and preserved differential renal function. Based on these findings and the absence of febrile UTIs, the initially planned surgery was cancelled. The patient remained asymptomatic, with negative urine cultures and stable renal function. Continuous antibiotic prophylaxis (CAP) was maintained until toilet training at three years of age, after which it was safely discontinued. By eight years of age, the ureterocele had remained stable, and the patient had no febrile UTIs or urinary symptoms.

This case illustrates that conservative management with sequential imaging can be a safe alternative to early surgery in asymptomatic infants. Our findings suggest that surgery should not automatically be performed at diagnosis but reconsidered in light of sequential imaging and renal function. In asymptomatic infants, an observation period of at least two to three months may be safely undertaken to evaluate the natural course. The combination of shrinkage, absence of infection, and preserved renal function supported long-term conservative management in this patient, contributing to defining safe criteria for conservative observation and CAP discontinuation.

## Introduction

A ureterocele is a cystic dilatation of the distal ureter, most often resulting from incomplete dissolution of the Chwalla membrane during embryogenesis [1]. The estimated incidence is approximately 1 in 4,000 live births, with a strong female predominance and bilateral occurrence in about 10% of cases [1-3]. Roughly 80% are associated with duplex systems, while single-system ureteroceles are less common [2]. Ureteroceles can be classified as intravesical or ectopic, with the latter more frequently linked to obstruction and symptomatic presentations. Asymptomatic cases are relatively uncommon but increasingly detected through routine prenatal ultrasound [3]. An ectopic ureterocele can be located distal to the trigone, inserted into the bladder neck, urethra, or elsewhere in the pelvis; it is often detected on imaging [1] and is frequently associated with vesicoureteral reflux (VUR) [2].

Although this anomaly can occur in a single-system kidney, it primarily involves the upper pole of a duplicated renal unit in approximately 80% of cases [4]. In approximately 40% of cases involving a sphincteric ureterocele, the ureteral orifice may be extravesical, which can either be of standard or enlarged size, extending into the bladder neck and opening anywhere proximal to the external sphincter. Several subtypes of ectopic ureteroceles have been described, including sphincteric ureteroceles, sphincter-stenotic ureteroceles, cecoureteroceles, and blind ectopic ureteral anomalies [5].

Patients with this condition may be at increased risk of urinary tract infections (UTIs), particularly when associated with VUR, obstruction, or duplicated systems. Although surgical interventions are frequently recommended, some patients can be managed with continuous antibiotic prophylaxis (CAP). Several case reports and small series in the literature have described conservative treatment for ectopic ureterocele [6,7], and recent reviews have also addressed this management option [8,9]. Several reviews have also addressed the potential role of conservative management and highlighted the ongoing debate regarding its indications and outcomes [10-12]. However, these reports remain limited, and the evidence base is heterogeneous. No consensus exists regarding the criteria for such treatment. Here, we report a rare case of ectopic ureterocele successfully managed conservatively, highlighting criteria for safe discontinuation of CAP.

## Case presentation

**Figure 1:**
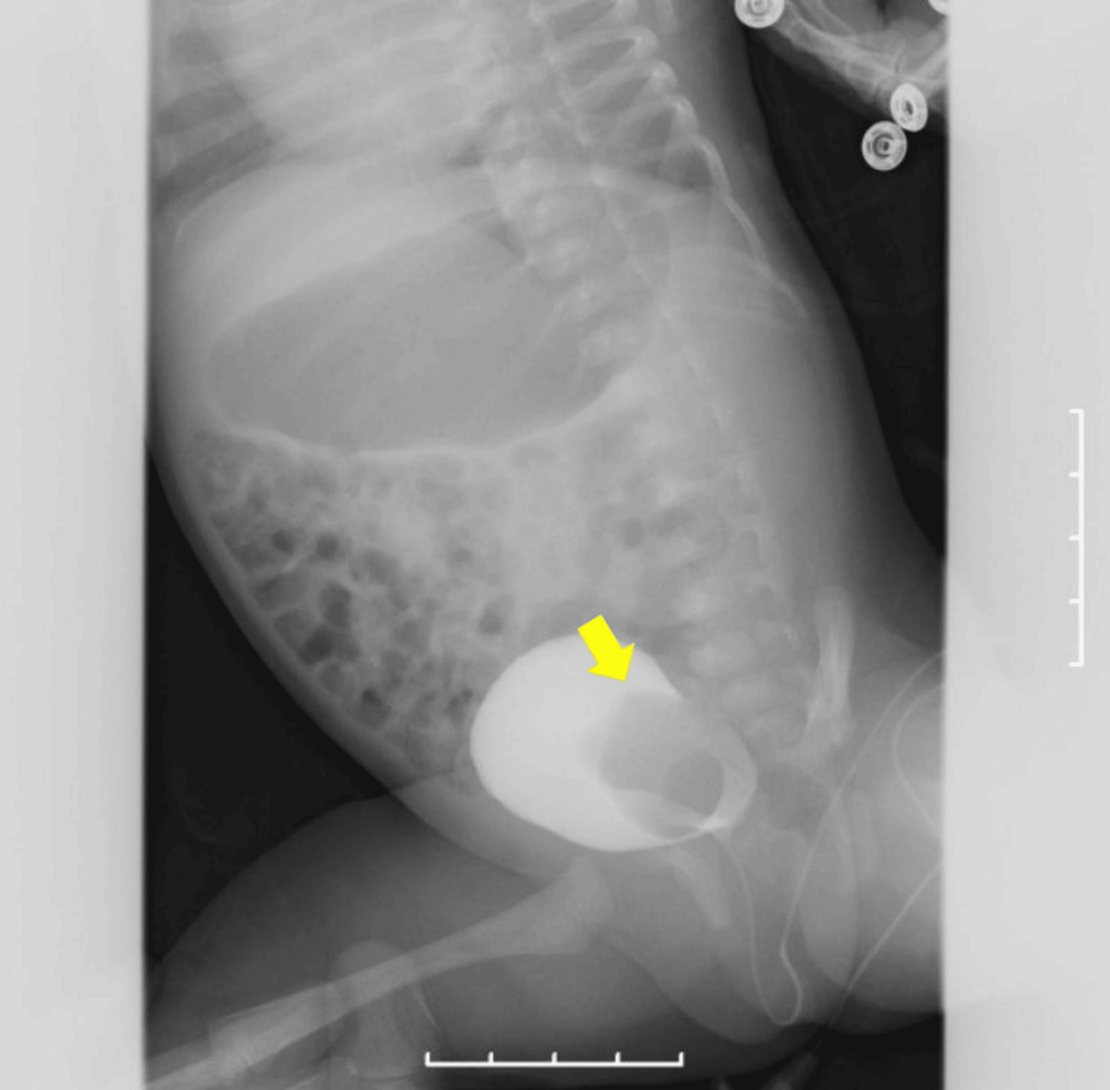
Voiding cystourethrography (VCUG) at one month of age. The arrow indicates the ureterocele located at the left ureteral orifice. No vesicoureteral reflux (VUR) was observed during the storage phase.

**Figure 2:**
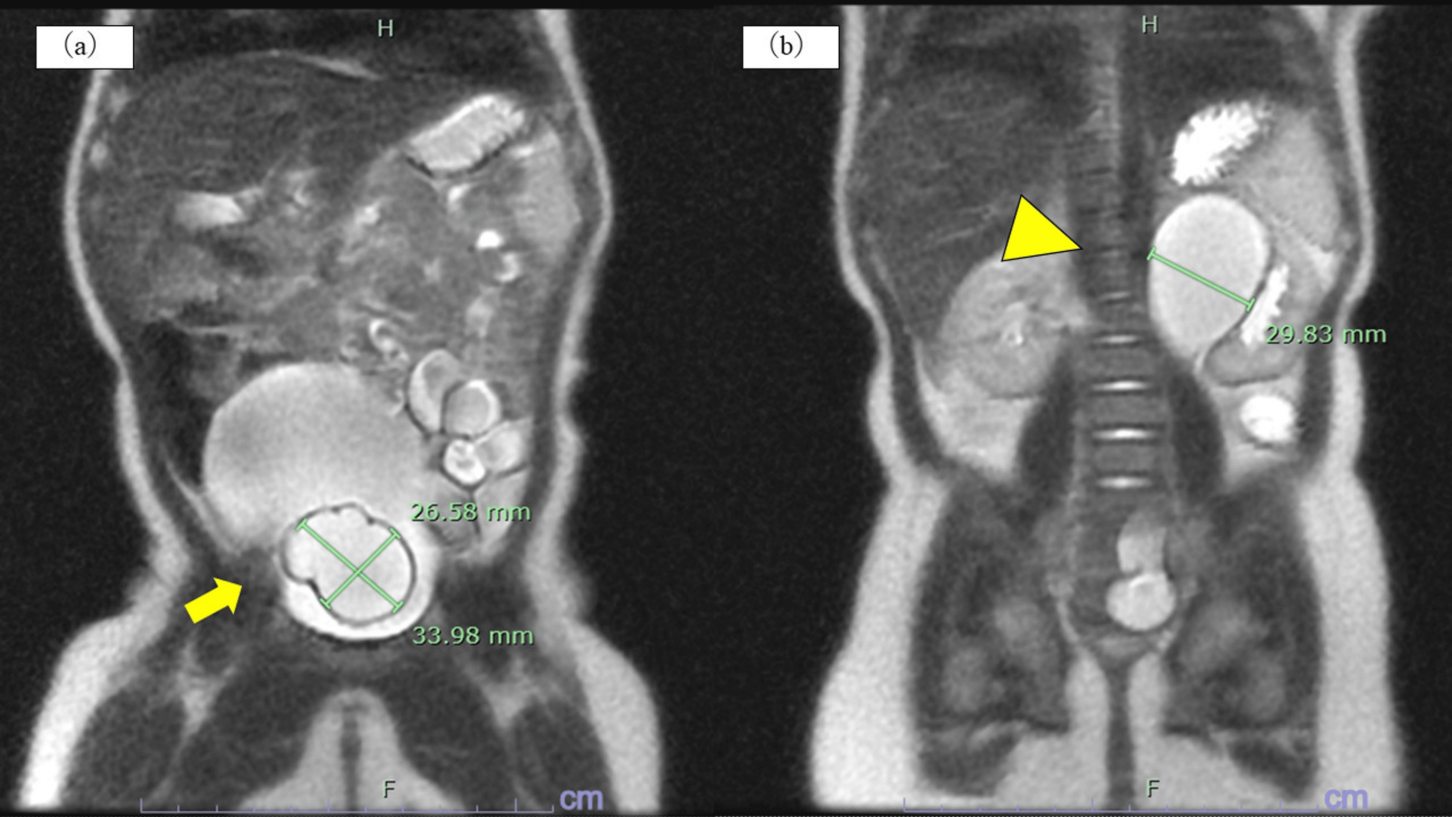
Magnetic resonance urography (MRU) at three months of age. (a) The arrow indicates the ureterocele measuring 33.98×26.58 mm in the bladder. (b) The triangle indicates the dilated left renal pelvis (29.83 mm).

**Figure 3:**
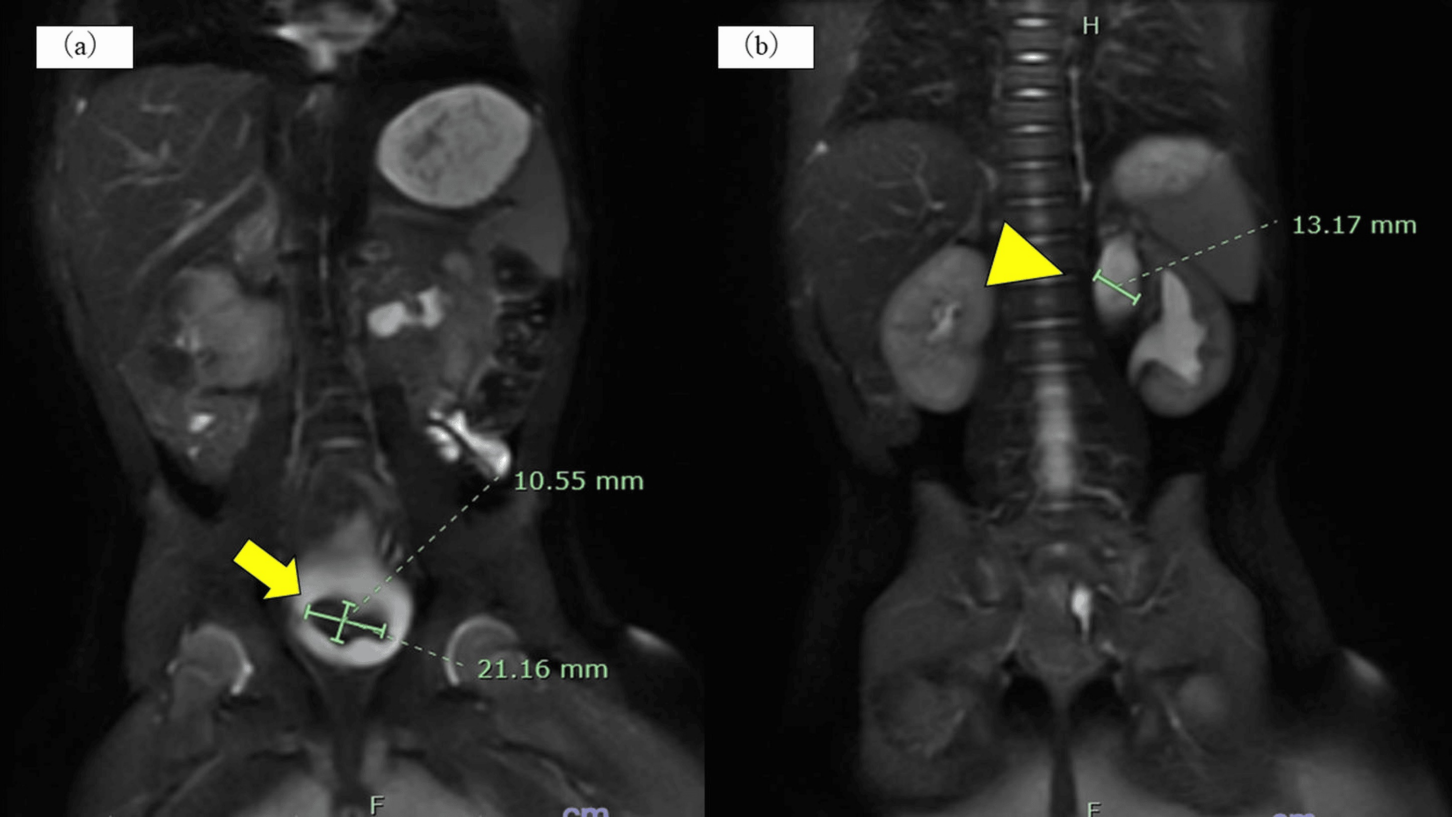
Magnetic resonance urography (MRU) at eight months of age. (a) The arrow indicates the reduced ureterocele (21.16×10.55 mm). (b) The triangle indicates improved hydronephrosis of the left upper kidney (13.7 mm).

**Figure 4:**
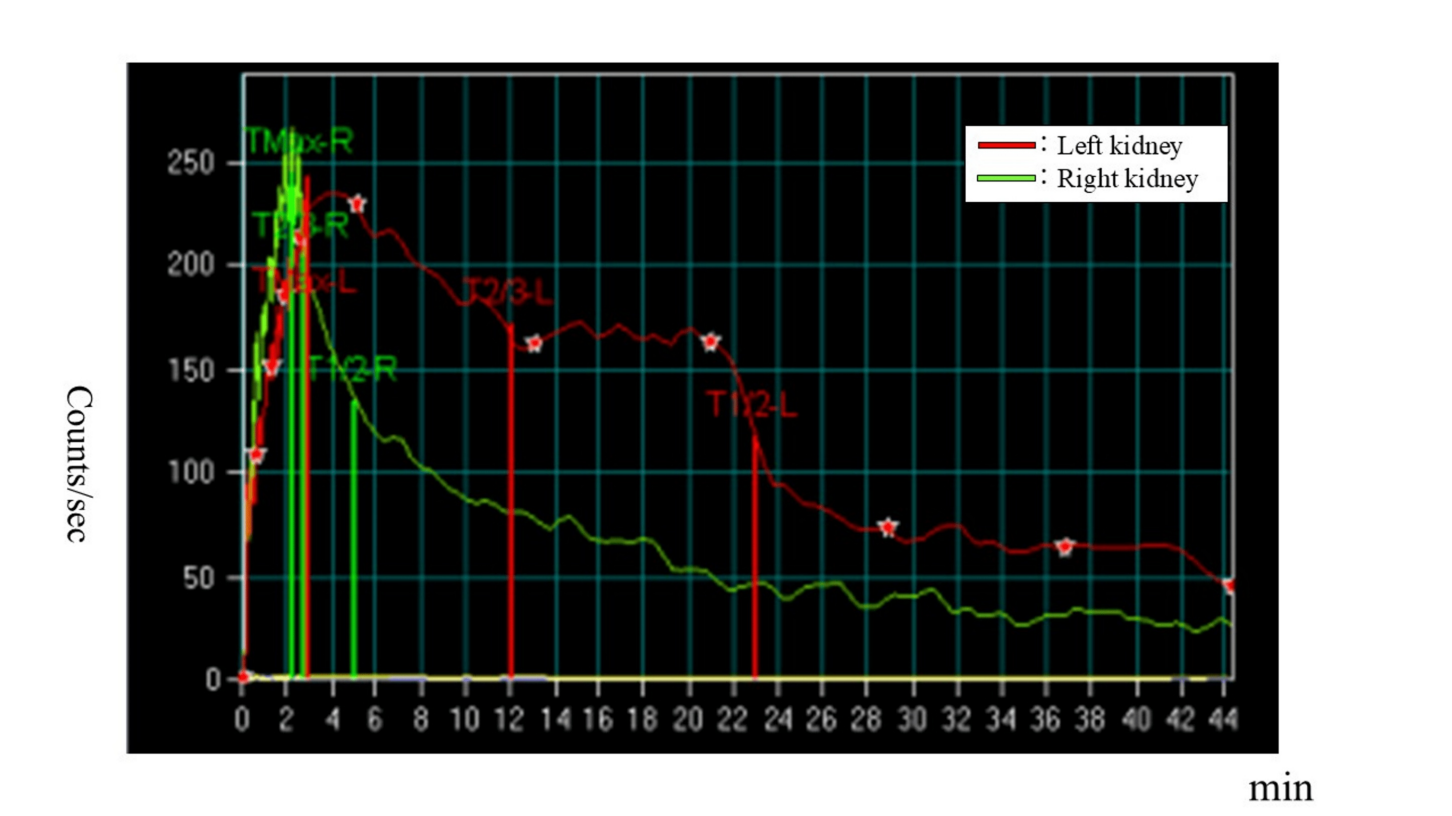
Mercaptoacetyltriglycine (MAG3) scintigraphy at eight months of age. Differential renal function (DRF) was preserved (42.6% left, 57.4% right). The red line is on the left kidney. The green line is on the right kidney.

**Figure 5:**
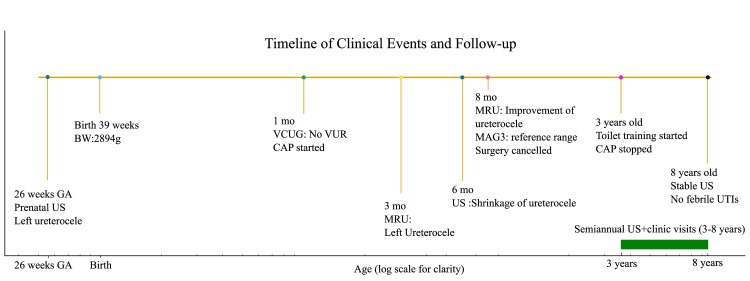
Timeline of this patient US, ultrasound; VCUG, voiding cystourethrography; VUR, vesicoureteral reflux; CAP, continuous antibiotic prophylaxis; MRU, magnetic resonance urography; MAG3, mercaptoacetyltriglycine scintigraphy; UTI, urinary tract infection.

A female infant was diagnosed prenatally with a left ureterocele during a routine fetal ultrasound at 26 weeks of gestation. She was delivered vaginally at 39 weeks and 0 days, with a birth weight of 2894 g. The perinatal course was uneventful. She had two healthy siblings, a seven-year-old brother and an 11-year-old sister, neither of whom had urinary tract anomalies. When the patient was one month old, postnatal ultrasound revealed left hydronephrosis and hydroureter. Voiding cystourethrography (VCUG) demonstrated a ureterocele in the bladder without VUR (Figure 1). Oral amoxicillin (50 mg/day) was initiated as CAP. At three months of age, magnetic resonance urography (MRU) identified a ureterocele measuring 33.98 × 26.58 mm at the left ureteral orifice. The duplicated renal pelvis and incomplete duplicated ureter were also dilated (29.83 mm) (Figure 2). Urine cultures obtained at this time were negative. Surgery (transurethral incision of the ureterocele, TUI-cele) was scheduled for eight months of age. By six months of age, routine ultrasound demonstrated shrinkage of the ureterocele, and the patient remained asymptomatic without fever or urinary symptoms. At seven months, urine culture was again negative. Follow-up MRU performed under anesthesia at eight months revealed further reduction in the ureterocele (21.16 × 10.55 mm) and improvement in hydronephrosis of the upper renal moiety (renal pelvis 13.7 mm) (Figure 3). Mercaptoacetyltriglycine (MAG3) scintigraphy showed preserved differential renal function (DRF: 42.6% left, 57.4% right) (Figure 4). Although hydronephrosis was present in the upper moiety, the lower moiety pelvis and ureter were carefully assessed on imaging and showed no dilatation at any time point. Therefore, systematic measurement of the lower moiety was not performed, and our quantitative follow-up focused on the upper moiety, which was the clinically affected system. There was no evidence of bladder neck or urethral obstruction. If obstruction had been present, ureteral dilatation would also have been expected, which was not observed. Given these results and the absence of febrile UTIs, the planned surgery was cancelled. Subsequent outpatient visits every three months and ultrasound every six months consistently demonstrated stable findings. When she reached three years, CAP was safely discontinued after toilet training was established. Thereafter, she continued follow-up every six months with ultrasound and blood tests, which confirmed stable ureterocele size, preserved renal morphology, and normal renal function. At eight years of age, the ureterocele had remained stable, renal function was preserved, and the patient had no urinary symptoms or febrile UTIs. A summary of quantitative follow-up findings, including ureterocele size, renal pelvis diameter, DRF, and UTI status at each time point, is provided in Table 1 and illustrated in the timeline (Figure 5).

**Table 1:** Quantitative follow-up data of the patient. The table summarizes ureterocele size, renal pelvis diameter, differential renal function (DRF), presence of vesicoureteral reflux (VUR), and urinary tract infection (UTI)/urine culture results at different time points during follow-up. Reference ranges: Ureterocele size has no defined normal value, as ureteroceles are abnormal findings. Renal pelvis diameter is generally not measurable in healthy infants; an anteroposterior diameter (APD) >10 mm is considered abnormal. Differential renal function (DRF) lacks an absolute cutoff, but values between 45 and 55% per kidney are typically regarded as normal. Age-specific ranges are not consistently available in the literature; therefore, representative values are provided here. N/A indicates data not assessed at that time point, as additional imaging was clinically unnecessary or deferred to avoid sedation and radiation exposure in an asymptomatic child.

Age	Findings/Imaging	Key Results	Management
26 weeks gestation	Prenatal ultrasound	Left ureterocele detected	Antenatal diagnosis
Birth (39 weeks)	Delivery (vaginal, BW 2894 g)	No perinatal complications; siblings healthy	Observation
1 month	Postnatal US, VCUG	Hydronephrosis, hydroureter; ureterocele in bladder; no VUR	CAP (amoxicillin) started
3 months	MRU	Ureterocele 33.98 × 26.58 mm; duplicated renal pelvis dilated (29.83 mm); urine culture negative	Surgery planned for eight months
6 months	Ultrasound	Ureterocele smaller; asymptomatic	Observation
8 months	MRU, MAG3	Ureterocele 21.16 × 10.55 mm; hydronephrosis improved (13.7 mm); DRF 42.6% left, 57.4% right; urine culture negative	Planned TUI-cele cancelled
3 years	Ultrasound, urine culture	Stable findings; no febrile UTI	CAP discontinued after toilet training
3–8 years	US + blood tests (6-monthly)	Stable ureterocele size, preserved renal morphology, normal renal function	Conservative management continued
8 years	Ultrasound + follow-up	Ureterocele stable; preserved renal function; no UTI	Long-term conservative management confirmed

## Discussion

Ectopic ureterocele has traditionally been managed with early surgical intervention, such as transurethral incision (TUI-cele), heminephrectomy, or ureteral reimplantation, particularly in the presence of febrile UTIs, progressive hydronephrosis, or impaired renal function [1].

Recent studies have suggested that conservative management may be considered in selected patients [6,7]. Our case emphasizes three important aspects.

First, surgical indication should be individualized. In our case, surgery (transurethral incision of the ureterocele, TUI-cele) was initially scheduled at eight months of age. This timing was chosen because endoscopic incision is generally considered feasible once infants reach sufficient body size for safe anesthesia and instrumentation, typically after six months of age [4].

Moreover, early intervention is usually reserved for patients with febrile UTIs or progressive renal impairment, whereas asymptomatic infants may be observed until later infancy to allow for potential spontaneous improvement [4,7]. Reassessment at six and eight months revealed ureterocele shrinkage, improvement in hydronephrosis, and preserved renal function. Together with the absence of febrile UTIs, these findings led us to cancel the planned surgery. This reinforces the principle that operative decisions should be re-evaluated after sequential imaging and functional studies rather than determined solely at diagnosis.

Second, our case illustrates the value of a defined observation window in asymptomatic infants. The patient was diagnosed antenatally, confirmed by VCUG at three months, and found to have ureterocele shrinkage on ultrasound at six months. Follow-up MRU and MAG3 at eight months confirmed further improvement, allowing surgery to be safely deferred. This suggests that in asymptomatic infants, an observation period of at least two to three months may be appropriate before definitive surgical decision-making.

Third, our case is consistent with previously reported experiences of conservative management. As summarized in Table 2, several reports have described spontaneous regression or stable long-term outcomes without surgical intervention [6]. These observations are in line with earlier reviews, which emphasized that while conservative management may be feasible in carefully selected cases, long-term outcomes are heterogeneous and the criteria for safe observation remain debated [10-12]. Table 2 provides an overview of these cases, highlighting clinical features, management strategies, and long-term outcomes. Favorable prognostic factors identified across these reports include the absence of high-grade vesicoureteral reflux, preserved DRF, and negative urine cultures under antibiotic prophylaxis.

**Table 2:** Reported cases of conservatively managed ectopic ureteroceles. The table summarizes published cases describing conservative management, including patient characteristics, vesicoureteral reflux (VUR) status, use of continuous antibiotic prophylaxis (CAP), follow-up duration, and outcomes. Favorable prognostic factors across these reports include absence of high-grade VUR, preserved differential renal function (DRF), and infection-free follow-up under CAP. Our present case is added for comparison.

Author, Year	Patients (n)	Age at Diagnosis	Vesicoureteral reflux (VUR)	Continuous antibiotic prophylaxis (CAP)	Follow-up Duration	Outcome
Maruo et al., 2020 (Japan) [6]	1	Antenatal	None	Yes	6 years	Ureterocele gradually shrank, no febrile UTI, no surgery required
Direnna et al., 2006 (Canada) [7]	10		4	Yes	1-11years	Complete resolution in 6 during a mean follow-up of 2 years.
Jain et al., 2021 (India) [8]	1	Infancy	None	Yes	5 years	Stable hydronephrosis, no febrile UTI, no surgery required
Thambidorai et al., 2015 (Malaysia) [13]	4	Neonates–Infants	Mixed	Mixed	3–6 years	Two patients conservatively managed with a stable outcome, two required surgery
Marei et al., 2025 (Review) [9]	Review	–	–	–	–	Summarized heterogeneous outcomes; conservative feasible in selected patients
Our case	1	Antenatal	None	Yes, stopped at 3 years	8 years	Ureterocele shrinkage, preserved renal function, no febrile UTIs, no surgery

In addition, our decision-making process was informed by published frameworks such as the Churchill classification [14], which categorizes ureteroceles according to anatomical and functional characteristics. While not universally applied, this system reinforces that conservative management may be appropriate in asymptomatic patients with preserved function and no high-grade reflux, consistent with our case. It should also be acknowledged that not all conservatively managed ureteroceles remain stable in the long term. Several published series have described patients who eventually required surgical intervention due to recurrent febrile UTIs, worsening hydronephrosis, or impaired renal function [4,8]. This suggests a potential publication bias, as successful conservative cases are more likely to be reported than unfavorable outcomes. Furthermore, heterogeneity exists across institutions in terms of patient selection, diagnostic protocols, and thresholds for surgery. These factors complicate direct comparison between reports and should be taken into account when interpreting the available evidence. Our case fulfilled several favorable criteria (absence of high-grade VUR, preserved renal function, spontaneous shrinkage, and negative urine cultures), which likely contributed to the safe avoidance of surgery and discontinuation of CAP.

Our report has two main limitations. First, the reference ranges used in Table 1 are generalized values. While normal measurements in pediatric patients may vary with age, detailed age-specific standards are not consistently reported in the literature. Therefore, representative cut-offs were applied. Second, long-term cross-sectional imaging such as MRU or MAG3 was not performed beyond infancy. Because the patient remained asymptomatic with stable renal function and ultrasound findings, additional investigations requiring prolonged anesthesia or exposing the child to radiation were not considered ethically or clinically necessary. Instead, the patient was monitored with clinical visits and semiannual ultrasound examinations, which consistently demonstrated stability and did not indicate a need for repeat sedated or invasive imaging. While this reflects common clinical practice, it limits the completeness of long-term imaging documentation.

Nevertheless, our case is distinctive in demonstrating the longest follow-up period reported to date - eight years - during which the patient remained infection-free, and surgery was avoided even after discontinuation of CAP.

Our review of the literature indicates that although the number of conservatively managed cases remains limited, the accumulation of long-term follow-up data, including our case, may help establish clinical criteria for observation. These findings, however, should be interpreted with caution and validated in larger patient cohorts.

Teaching points

Quantitative imaging follow-up (ureterocele size, renal pelvis diameter, differential renal function) is essential for objective assessment of conservative management. Absence of VUR, negative urine cultures, and preserved renal function support observation rather than immediate surgery. CAP discontinuation can be considered after toilet training if the patient has remained infection-free and imaging findings have improved. Long-term follow-up into school age provides strong evidence for the safety of conservative management in selected cases.

## Conclusions

This case demonstrates that an ectopic ureterocele can be safely managed conservatively when the patient remains asymptomatic, the ureterocele progressively decreases in size, renal function is preserved, and no VUR or UTI is present. CAP may be discontinued once toilet training is established and infection-free status is confirmed. This observation should be interpreted as a preliminary suggestion requiring validation in larger cohorts.
